# Gait rehabilitation machines based on programmable footplates

**DOI:** 10.1186/1743-0003-4-2

**Published:** 2007-02-09

**Authors:** Henning Schmidt, Cordula Werner, Rolf Bernhardt, Stefan Hesse, Jörg Krüger

**Affiliations:** 1 Department of Automation and Robotics, Fraunhofer IPK, Pascalstrasse 8-9, 10587 Berlin, Germany; 2 Department of Neurological Rehabilitation, Charité University Hospital, Kladower Damm 223, 14089 Berlin, Germany

## Abstract

**Background:**

Gait restoration is an integral part of rehabilitation of brain lesioned patients. Modern concepts favour a task-specific repetitive approach, i.e. who wants to regain walking has to walk, while tone-inhibiting and gait preparatory manoeuvres had dominated therapy before. Following the first mobilization out of the bed, the wheelchair-bound patient should have the possibility to practise complex gait cycles as soon as possible. Steps in this direction were treadmill training with partial body weight support and most recently gait machines enabling the repetitive training of even surface gait and even of stair climbing.

**Results:**

With treadmill training harness-secured and partially relieved wheelchair-mobilised patients could practise up to 1000 steps per session for the first time. Controlled trials in stroke and SCI patients, however, failed to show a superior result when compared to walking exercise on the floor. Most likely explanation was the effort for the therapists, e.g. manually setting the paretic limbs during the swing phase resulting in a too little gait intensity. The next steps were gait machines, either consisting of a powered exoskeleton and a treadmill (Lokomat, AutoAmbulator) or an electromechanical solution with the harness secured patient placed on movable foot plates (Gait Trainer GT I). For the latter, a large multi-centre trial with 155 non-ambulatory stroke patients (DEGAS) revealed a superior gait ability and competence in basic activities of living in the experimental group. The HapticWalker continued the end effector concept of movable foot plates, now fully programmable and equipped with 6 DOF force sensors. This device for the first time enables training of arbitrary walking situations, hence not only the simulation of floor walking but also for example of stair climbing and perturbations.

**Conclusion:**

Locomotor therapy is a fascinating new tool in rehabilitation, which is in line with modern principles of motor relearning promoting a task-specific repetitive approach. Sophisticated technical developments and positive randomized controlled trials form the basis of a growing acceptance worldwide to the benefits or our patients.

## Background

The restoration of gait for patients with impairments of the central nervous system (CNS), like e.g. stroke, spinal cord injury (SCI) and traumatic brain injury (TBI) is an integral part of rehabilitation and often influences whether a patient can return home or to work. Particularly stroke is the leading cause for disability in all industrialized countries, the incidence is approximately one million patients in the European Union each year [1,2]. Modern concepts of motor learning favor a task specific training, i.e. to relearn walking, the patient should ideally train all walking movements, needed in daily life, repetitively in a physically correct manner [3]. Conventional training methods based on this approach, proved to be effective, e.g. treadmill training [4], but they require great physical effort from the physiotherapists to assist the patient, so does even more training of free walking guided by at least two physiotherapists. Assisted gait movements other than walking on even floor, like for instance stair climbing, are practically almost impossible to train, due to the overstrain of the physiotherapists. Assistive training devices, in particular those based on the concept of programmable footplates, may offer a solution to these shortcomings.

## Gait Rehabilitation

Hemiparesis is the typical sequelae following stroke, three months after the incident one third of the surviving patients has not yet regained independent walking ability, and those ambulatory walk in a typical asymmetric manner, as they avoid to load the paretic limb. At the same time their walking velocity and endurance are markedly reduced. Stairs, sudden obstacles, uneven terrain or other perturbations further challenge the patients' gait ability outside the clinic. The rehabilitation process toward regaining a meaningful mobility can be divided into three phases [5]:

1. the bedridden patient has to be mobilized into the wheelchair,

2. restoration of gait,

3. and improvement of gait in order to meet the requirements of daily mobility.

For the *first phase*, an early mobilization policy is generally accepted, i.e. over the edge of the bed the patient is transferred into the chair as soon as possible. The *second phase*, restoration of gait, has seen major changes in the last decade, in particular with the introduction of a task-specific repetitive gait training approach. During this phase gait rehabilitation machines come into play, especially for severely affected patients. They relieve the physiotherapists from hard manual labour and enable an increase in training intensity for the patients, the latter is an important factor in motor learning. Until the beginning of the *third phase *patients have regained walking ability and can further improve it now by training of free walking. Depending on the patients learning success, support by physiotherapists may still be necessary during this phase. The following chapters mainly refer to the second phase.

### Rehabilitation Concepts

Proponents of so called *neurophysiological treatment concepts *(Bobath, PNF, Brunnstroem, Vojta) aimed at the restoration of a most physiological gait pattern [6]. Bobath therapists, the most widely accepted treatment concept in Europe, intended to inhibit an increased muscle tone (spasticity) by gently mobilizing the paretic limbs and opposing synergistic movements, and to repeat quasi in short form the statomotoric development of a child as prerequisite for the final goal of a most natural walking habit. Accordingly, tone-inhibiting manoeuvres and motor tasks while lying, sitting or standing dominated therapy sessions of patients, who desperately wished to walk. Some therapists even recommended to sit again into the wheelchair being afraid of the patient familiarizing with his bad gait quality.

This policy collided with modern *task-specific repetitive concepts *of motor learning, emerging in the early nineties, i.e. who wants to relearn walking has to walk. Locomotor therapy by treadmill training with partial body weight support was a first step in this direction. The patient wore a harness to substitute for deficient equilibrium reflexes, part of his body weight was relieved to compensate for the paresis of the impaired lower limb, and the motor-driven treadmill enforced locomotion [7,8]. Wheelchair-bound patients could thus practice up to 1000 steps during a 30 min session as compared to 50 to 100 at maximum during a conventional therapy session. Two therapists assisted the patients' gait, sitting alongside to place the paretic limb, to ensure an initial contact with the heel, to prevent a knee hyperextensor thrust and to control for a symmetric step length. Standing behind the patient, a second therapist shifted the weight according to stance/swing phase, promoted hip extension and trunk erection. The concept of locomotor therapy was striking: massive gait practice to activate spinal and supraspinal pattern generators and an efficient cardiovascular training of the deconditioned and often multimorbide patients.

### Clinical Studies

A major clinical study in order to investigate the locomotor therapy concept, was an A-B-A study (A: treadmill, B: physiotherapy, each phase lasted 3 weeks) which showed that the chronic non-ambulatory stroke patients exclusively improved their gait ability and walking velocity during the A-phases [8]. Those results clearly supported the task-specific training concept, as the physiotherapy had followed a very conservative Bobath approach with the practice of gait itself minimal.

Subsequent RCTs in acute non-ambulatory patients accordingly compared treadmill training with gait practice on the floor. The results were unexpected: treadmill training and gait practice did not differ with respect to the restoration of gait, and a Cochrane meta analysis came to the conclusion that locomotor therapy on the treadmill was not superior [9]. What had happened? Due to the comparable effort for the therapists assisting the patients' gait on the treadmill and on the floor, the number of steps practiced probably had not differed between the two conditions. Unfortunately neither article reported exact numbers, but it was not uncommon that therapists stopped the treadmill treatment after 300 to 400 steps due to fatigue.

## Gait Rehabilitation Machines

**Figure 1:**
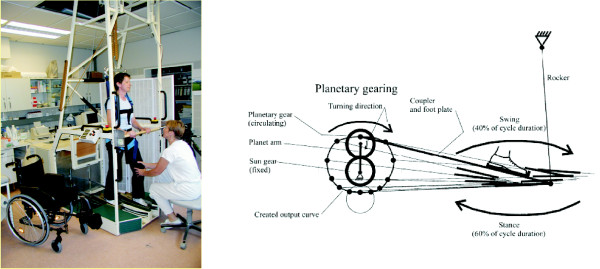
**Electromechanical Gait Trainer GT I with movable footplates**. The photograph on the left shows gait rehabilitation with stroke patient, the technical sketch on the right shows the functional principle of the machine.

The development of gait rehabilitation devices started with machines for training of walking on even ground, beginning with the electromechanical 'Gait Trainer GT I' developed by our group [10] (see Fig. 1) and the Driven Gait Orthosis (DGO) 'Lokomat', an exoskeleton type robot in combination with a treadmill developed by a group from University Hospital Balgrist/ETH Zurich [11].

Until now three other prototypes using the latter approach were designed [12-14]. The machines allow more effective training sessions, where patients can train up to 1000 steps within a typical training session of 15–20 min, whereas during manually assisted training only approx. 100 steps/session were performed. A second major effect is the relief of the physiotherapists, who can now concentrate on training supervision.

### Machine Concepts

The aforementioned machines apply two different approaches to gait rehabilitation: the *exoskeleton type machines*, which need to be operated in combination with a treadmill. They require the patient to be fixed to the robot kinematics from the pelvis on downward along the legs, which results in a bilateral and proximal guidance. The patients body weight is carried by the treadmill. Due to the complete fixture of the patient to the machine, the device is not designed for physical access of the therapist during the training session, but for fully automated training sessions.

In contrast the Gait Trainer GT I applies the principle of *movable footplates*, where each of the patients feet is positioned on a separate footplate whose movements are controlled by a planetary gear system, simulating foot motion during stance and swing. Cadence and stride length could be set individually. Further, the vertical and horizontal movements of the centre of mass were controlled via ropes attached to the harness. This machine concept leads to a bilateral and distal guided gait training. The patients knees are not fixed, in order to allow the therapists access for physical contact with the patient, which is an important factor in rehabilitation [2,15] and also allows him to do minor corrections of the knee motion if needed. Alternatively other techniques to stabilize knee motion like Functional Electrical Stimulation (FES) or separate mechanical fixtures can be applied if necessary. We optionally used a programmable 8-channel FES system enabling the individually adjusted stimulation of lower limb muscles, in order to control the paretic knee or to assist push-off during the terminal stance phase. Another important reason for this design approach is that the   number of constraints on natural hip and leg motion (muscles and joints)   and the large number of degrees of freedom of the human leg should be   kept as low as possible. The tighter the attachment of the leg to a   robot arm with a technically limited number of degrees of freedom, the   more constrained the resulting leg motion.

### Clinical Studies

**Figure 2:**
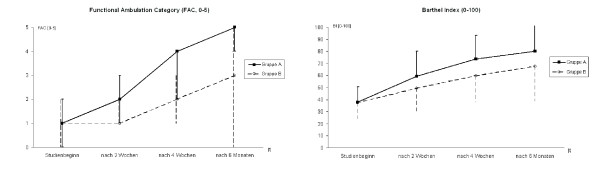
**DEGAS multi-center-RCT results comparing GT I based training with conventional gait training (Group A: GT I + PT, Group B: PT only).** The diagram on the left shows the Functional Ambulation Category score (FAC, 0–5), the diagram on the right shows the Barthel Index (0–100).

Several clinical studies with both types of machines have been done [16,17], the largest clinical study for gait rehabilitation machines worldwide was the DEGAS (DEutsche GAngtrainer Studie = German Gait Trainer Study) study, which was published recently [19]. DEGAS was a multi-center RCT study in which more than 150 stroke patients at four different German rehabilitation hospitals were involved. The study compared machine supported training (GT I) and conventional gait training (PT), thus reflecting common daily practice of a combination of locomotor and physiotherapy. Hence the setup compared 20 min of GT I + 25 min PT vs. 45 min PT every day for 4 weeks in non-ambulatory subacute stroke patients. The DEGAS results revealed a superior gait ability (Functional Ambulation Category, FAC 0–5 [20,21]) and competence in activities of daily living (ADL, Barthel Index 0–100 [22]) in the experimental group, the favorable effects persisted 6 months later. Figure 2 shows the major results of the DEGAS study.

To the best of our knowledge, no other gait rehabilitation device could achieve comparable results, even though in the DEGAS study as well as in all other GT I studies the machine was run in position controlled mode.

Why did the gait trainer lead to a better outcome than the manually assisted treadmill in previous RCTs? Again, training intensity was the most likely explanation: on the GT I the patients could continuously practice 800 to 1000 steps per session, as the effort of the therapists was drastically reduced. RCTs in Slovenia [23], Korea [24], and Hong Kong [25] confirmed the DEGAS results for acute non-ambulatory stroke patients. For chronic, ambulatory patients a Finnish study [26] revealed that an intensive gait training either on the machine or on the floor and even outside the clinic were equally effective. These findings support the intensity principle as ambulatory patients were encouraged to train as many steps as possible in their conventional physiotherapy sessions.

## Programmable Footplate based Gait Rehabilitation Robot

**Figure 3:**
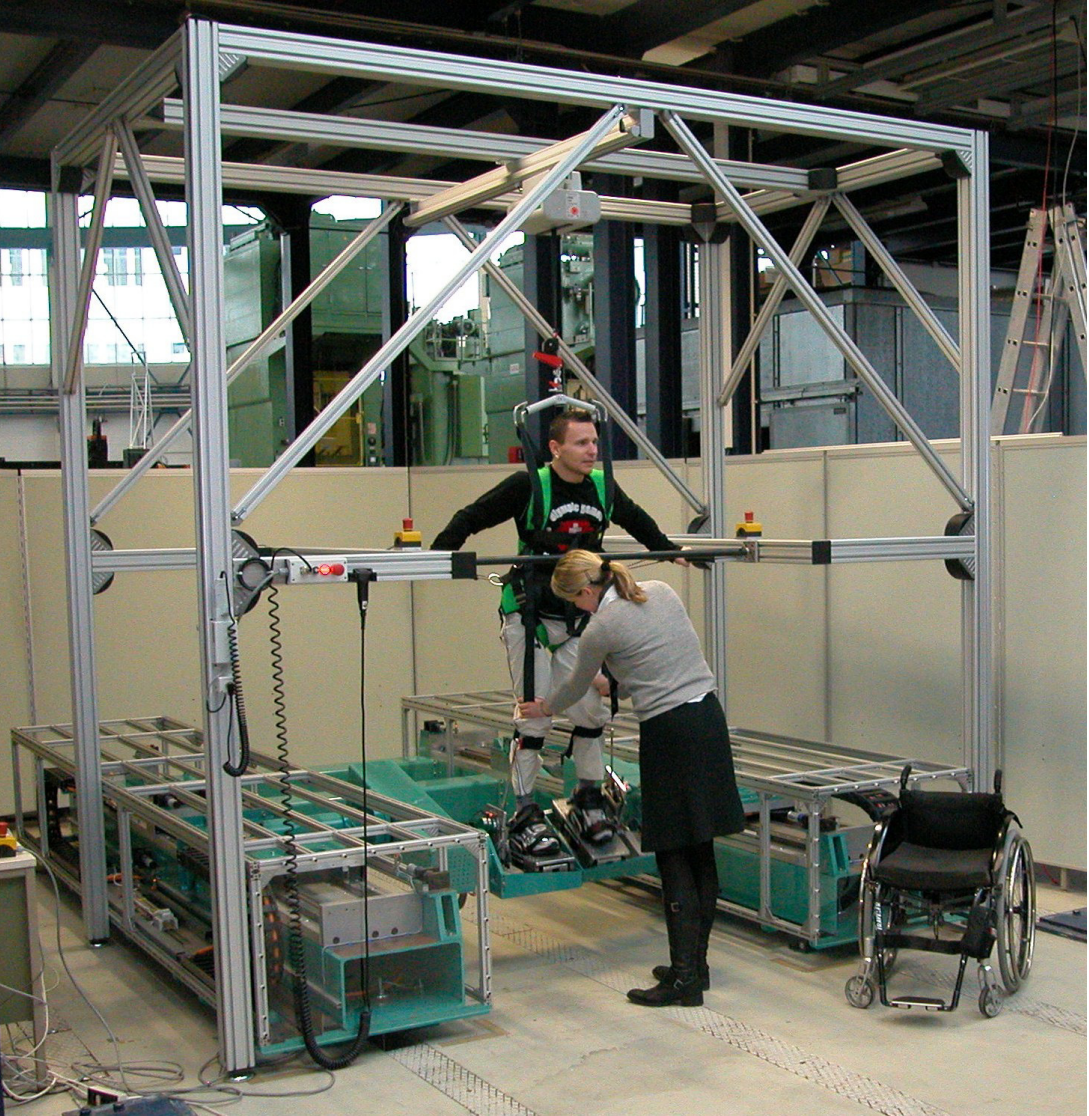
**HapticWalker with SCI patient and physiotherapist**. Photograph of the robotic walking simulator for gait rehabilitation HapticWalker.

A major problem in rehabilitation and motor learning is, that the transfer of learning from one motion pattern to a different one (e.g. transfer from walking on even ground to stair climbing) motion is very limited [2,27-29]. Hence the DEGAS results could lead to stay idle, but a patient, who was ambulatory on the floor (that was the criterion of an independent gait), could still prefer a wheelchair in daily life, as stair climbing and mastering sudden perturbations could have demanded too much of him. In order to satisfy the task specific approach paradigm for motor rehabilitation, it would be necessary to train as many different daily life walking situations as possible during gait rehabilitation. So far, an early stair climbing therapy requires the help of up to three therapists, and perturbations (e.g. stumbling, sliding) can hardly be mimicked in a clinical setting of an in-patient rehabilitation. Therefore the group decided to extend the successfully applied machine concept of movable footplates to a device comprising freely programmable footplates. This required the development of a new robotic gait rehabilitation device, named HapticWalker (see Fig. 3), which is based on the principle of programmable footplates. On such a machine the footplates are mounted at the end effectors of two separate robot arms.

The HapticWalker accomplishes the paradigm for optimal training, because it is the first gait rehabilitation device which is not restricted to training of walking on even ground. In contrast to all treadmill bound machines, it enables the patient to train arbitrary gait trajectories and daily life walking situations. It is also distinct from the small number of haptic foot device prototypes, which have been built by groups in the USA [30,31] and Japan [32,33] for healthy subjects (e.g. virtual soldier training). Unlike these machines, which are designed to provide contact between foot and footplate only during stance phase, the HapticWalker comprises a translatory and rotatory footplate workspace needed for permanent foot attachment along arbitrary walking trajectories during all phases of gait. This is an essential feature for gait rehabilitation machines. A group at Rutgers University [34] recently built a small walking simulator testbed with permanent foot attachment based on two small Stuart platforms. Those motion platforms, which are also called hexapod platforms, are based on a parallel kinematics principle and often used for flight simulators. The workspace and dynamic range of the small Stewart platforms the group designed are very limited and do not allow for natural walking profiles.

The HapticWalker footplate dynamics were designed such that not only smooth foot motions at moderate walking speeds can be accomplished, but also the realistic simulation of walking speeds of up to 5 km/h and 120 steps/min. This takes into account different gait rehabilitation strategies, the most widely practised starts with walking speeds of less than 1 km/h and gradually increases gait speed and cadence up to normal walking velocities of 4–5 km/h depending on the patients learning success [2]. In contrast some clinical groups in the US [35] favor the application of high walking speeds and cadences of 4–5 km/h right from the very beginning of therapy. The purpose of high footplate dynamics was also to enable the realistic simulation of gait perturbations like stumbling, sliding and other asynchronous walking events. Regarding usability, the HapticWalker is designed to allow therapists access to the patient for physical contact during training from all sides, as well as facilities for easy patient transfer into the machine, since they are usually bound to the wheelchair. Technical details of the machine design, robot kinematics, control system, algorithms for motion generation, haptic features, therapist user interface and safety aspects can be found in [36] and the referring references cited in there.

## Conclusion

Efficient gait rehabilitation requires the CNS impaired patient to practise as many different daily life walking trajectories as intensive as possible. The HapticWalker, a generic robotic walking simulator based on the principle of programmable footplates, is the first device to fulfil these requirements by allowing the training of arbitrary walking situations and foot trajectories (e.g. even ground, stair climbing up/down, perturbations like stumbling/sliding). The task specific gait rehabilitation concept of repetitive foot motions on natural trajectory profiles was proved by different clinical research groups worldwide. Our group coordinated the DEGAS study, the largest clinical multi-center RCT study for gait rehabilitation machines worldwide. It investigated the movable footplate based electromechanical Gait Trainer GT I including its position controlled, bilateral and distal approach compared to conventional gait training. The study was finished recently and fully proved the definitive advantages and benefits of this gait rehabilitation approach for the patients. The robotic gait trainer HapticWalker extends this concept to programmable footplates, thus it optimally fulfills the requirements of the task specific training paradigm. A full working prototype of the Haptic Walker was successfully developed and built, it is currently being clinically tested after receiving full approvals by the German Technical Committee for Medical Devices (TÜV) and the Charité ethics board. A clinical study with focus on staircase walking in addition to even ground walking was started in order to evaluate the machine. First trials with stroke and SCI patients are very promising and give reason to anticipate even better results than the ones seen in the DEGAS study.
